# Supplementary material for: Synthesis, Antimicrobial, Anticancer, PASS, Molecular Docking, Molecular Dynamic Simulations & Pharmacokinetic Predictions of Some Methyl β-D-Galactopyranoside Analogs
Source: Molecules. 2021 Nov 20;26(22):7016. doi: 10.3390/molecules26227016 (PMC8621697; doi:10.3390/molecules26227016)
Supplement: Supplementary file 1 [file molecules-26-07016-s001.zip › molecules-1421643-supplementary.pdf]

molecules

Article

Synthesis, Antimicrobial, Anticancer, PASS, Molecular Docking, Molecular Dynamic Simulations & Pharmacokinetic Predictions of some Methyl β-D-Galactopyranoside Analogs

Md. Ruhul Amin[1], Farhana Yasmin[1], Mohammed Anowar Hosen[1], Sujan Dey[2], Shafi Mahmud[3], Md. Abu Saleh[3], Talha Bin Emran[4], Imtiaj Hasan[5], Yuki Fujii[6], Masao Yamada[7], Yasuhiro Ozeki[7,*] and Sarkar Mohammad Abe Kawsar[1,*]

[1]Laboratory of Carbohydrate and Nucleoside Chemistry, Department of Chemistry, Faculty of Science, University of Chittagong, Chittagong-4331, Bangladesh

[2]Department of Microbiology, Faculty of Biological Science, University of Chittagong, Chittagong 4331, Bangladesh

[3]Microbiology Laboratory, Department of Genetic Engineering and Biotechnology, University of Rajshahi, Rajshahi 6205, Bangladesh

[4]Department of Pharmacy, BGC Trust University Bangladesh, Chittagong-4381, Bangladesh

[5]Department of Biochemistry and Molecular Biology, Faculty of Science, University of Rajshahi, Rajshahi 6205, Bangladesh

[6]School of Pharmaceutical Sciences, Nagasaki International University, 2825-7, Huis Ten Bosch-cho, Sasebo, Nagasaki 859-3298, Japan

[7]School of Sciences, Yokohama City University, 22-2, Seto, Kanazawa-ku, Yokohama 236-0027, Japan

* Correspondence: akawsarabe@yahoo.com (SMAK); ozeki@yokohama-cu.ac.jp (YO.)

Citation: Amin, M.R.; Yasmin, F.; Hosen, M.A.; Dey, S.; Mahmud, S.; Saleh, M.A.; Emran, T.B.; Hasan, I.; Fujii, Y.; Yamada, M.; Ozeki, Y.; Kawsar, S.M.A. Synthesis, Antimicrobial, Anticancer, PASS, Molecular Docking, Molecular Dynamic Simulations & Pharmacokinetic Predictions of Some Methyl β-D-Galactopyranoside Analogs. *Molecules* **2021**, *26*, 7016. https://doi.org/10.3390/molecules26227016

Academic Editor: Firstname Lastname

Received: date

Accepted: date

Published: date

Publisher's Note: MDPI stays neutral with regard to jurisdictional claims in published maps and institutional affiliations.

Copyright: © 2021 by the authors. Submitted for possible open access publication under the terms and conditions of the Creative Commons Attribution (CC BY) license (https://creativecommons.org/licenses/by/4.0/).

Supplementary Materials

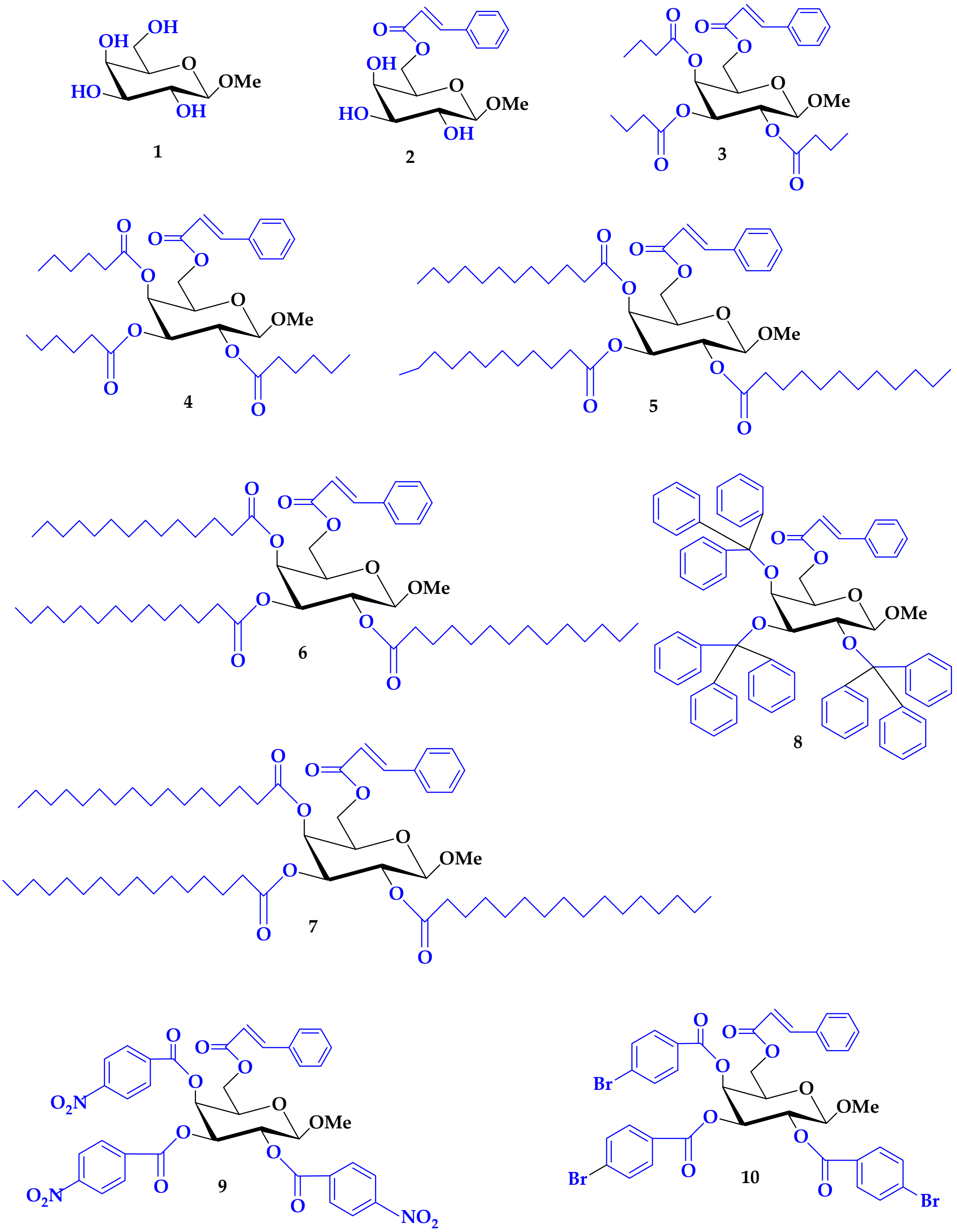

Figure S1. 2D structure of designed MGP derivatives (**2-10**).

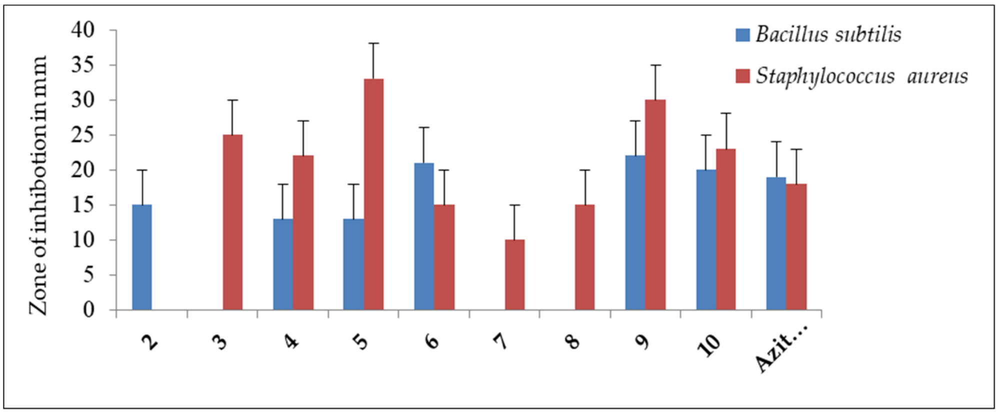

Figure S2. Zone of inhibition was observed against Gram-positive bacteria by the tested analogs.

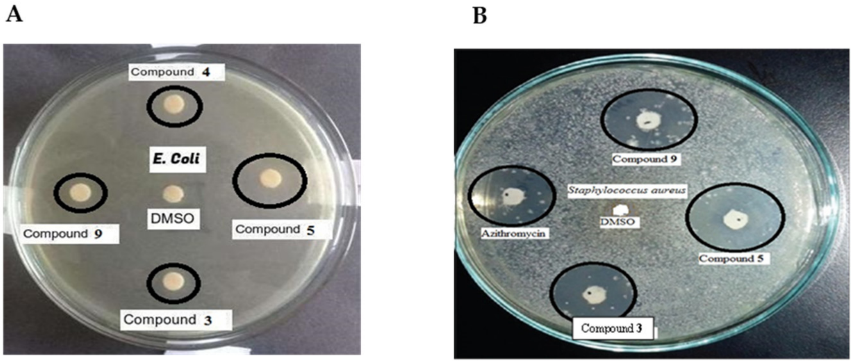

Figure S3. Some experimental dishes of the synthesized test analogs against bacteria. **A** against *E. coli* by four test analogs **3, 4, 5,** and **9; B.** against *S. aureus* by test analogs **5, 9,** and **3.** Here DMSO = negative control and Azithromycin = positive control.

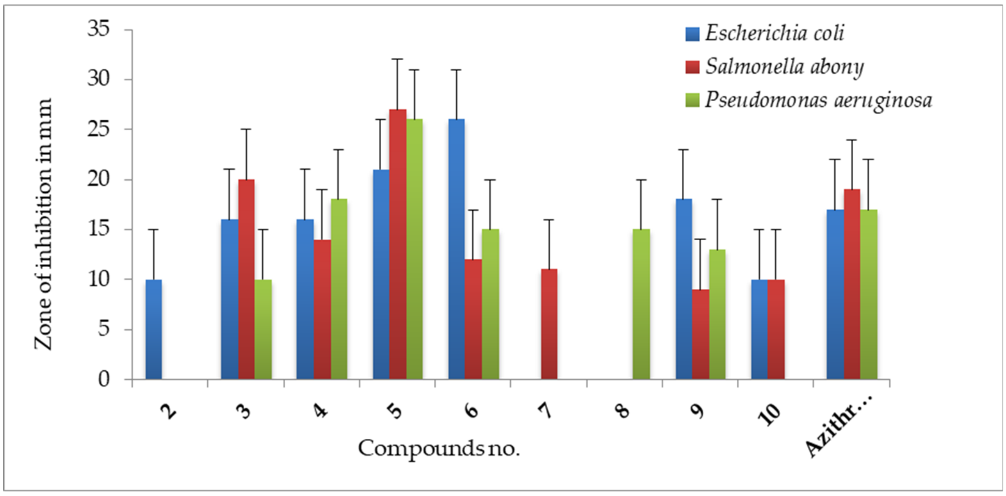

Figure S4. Zone of inhibition observed against Gram-negative bacteria by the tested analogs.

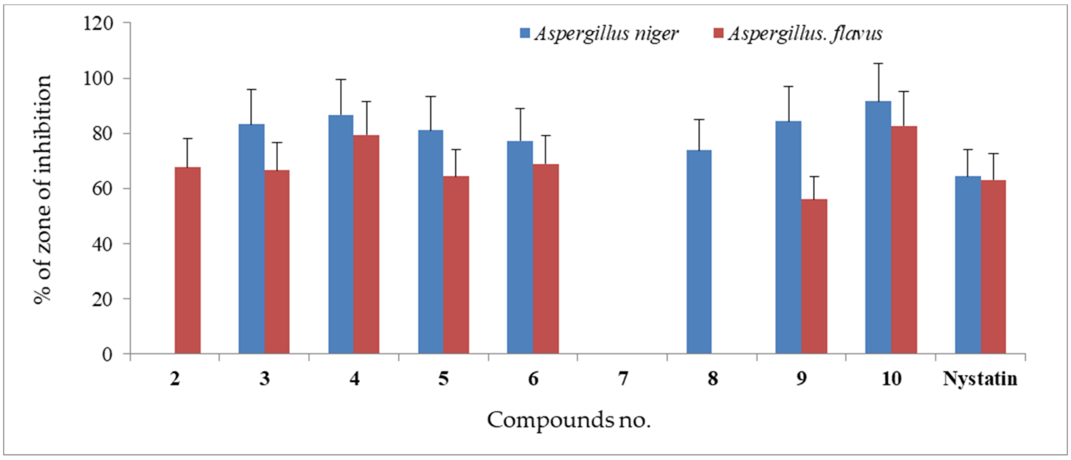

Figure S5. Antifungal activities of the synthesized test analogs.

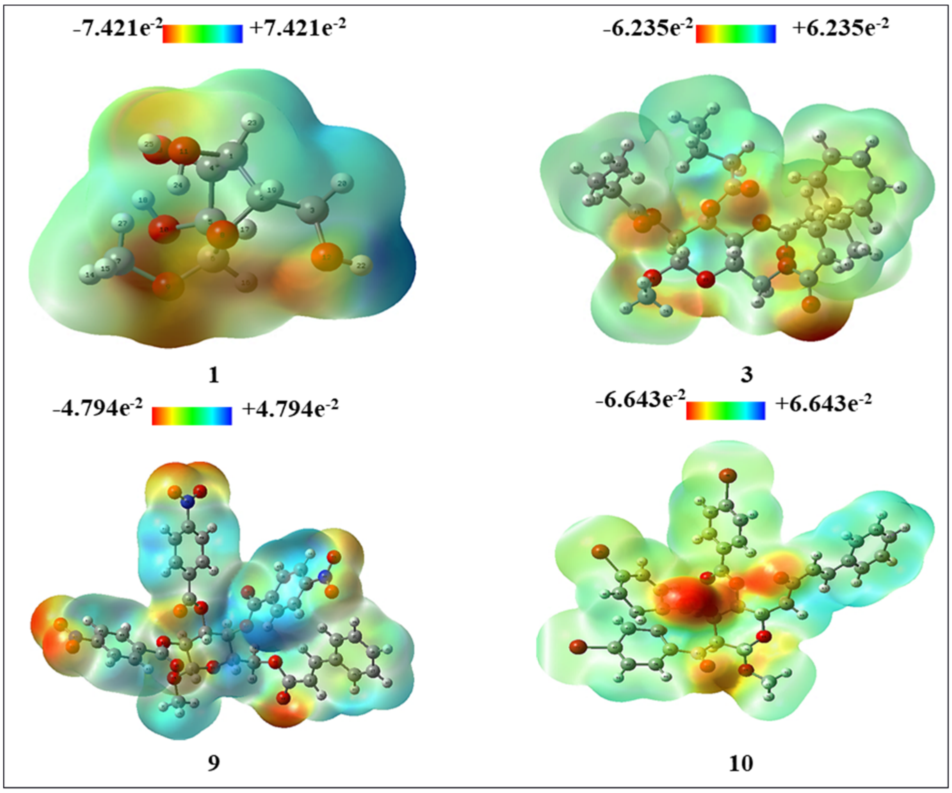

Figure S6. Map of the molecular electrostatic potential of MGP (**1**) and analogs (**3, 9,** and **10**).

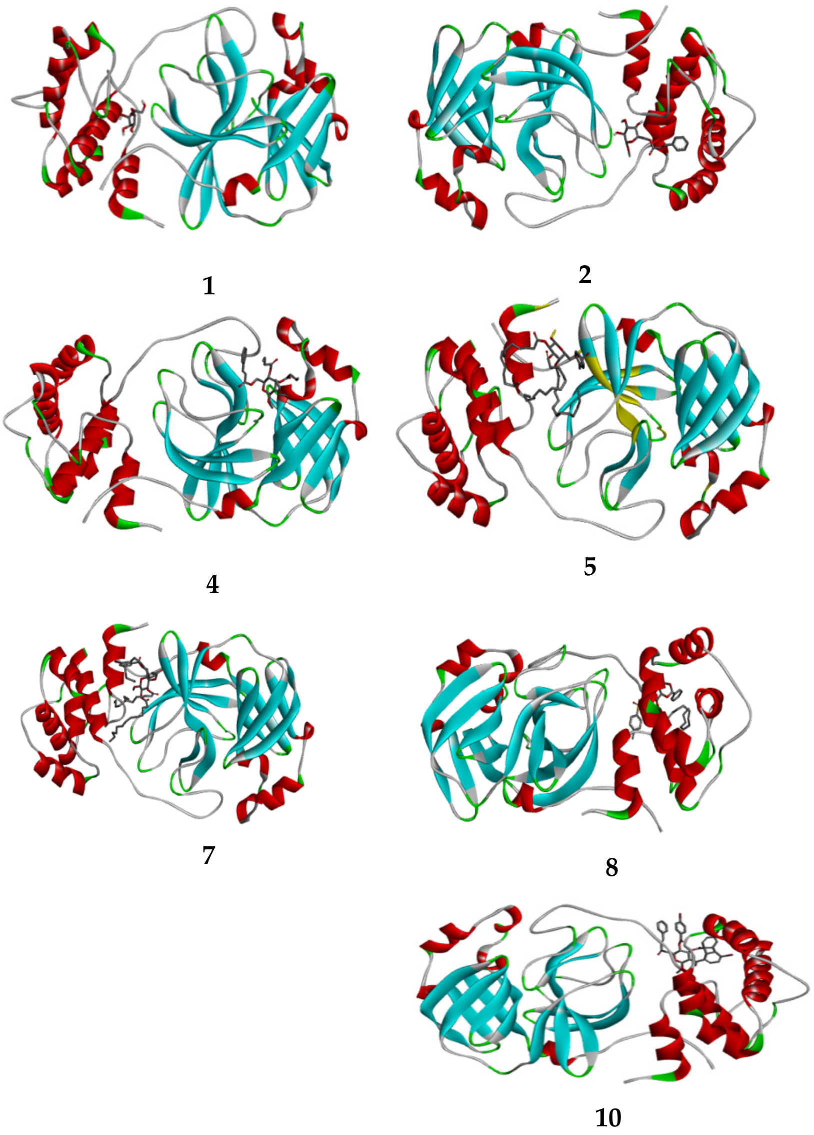

Figure S7. Docked view of all compounds.

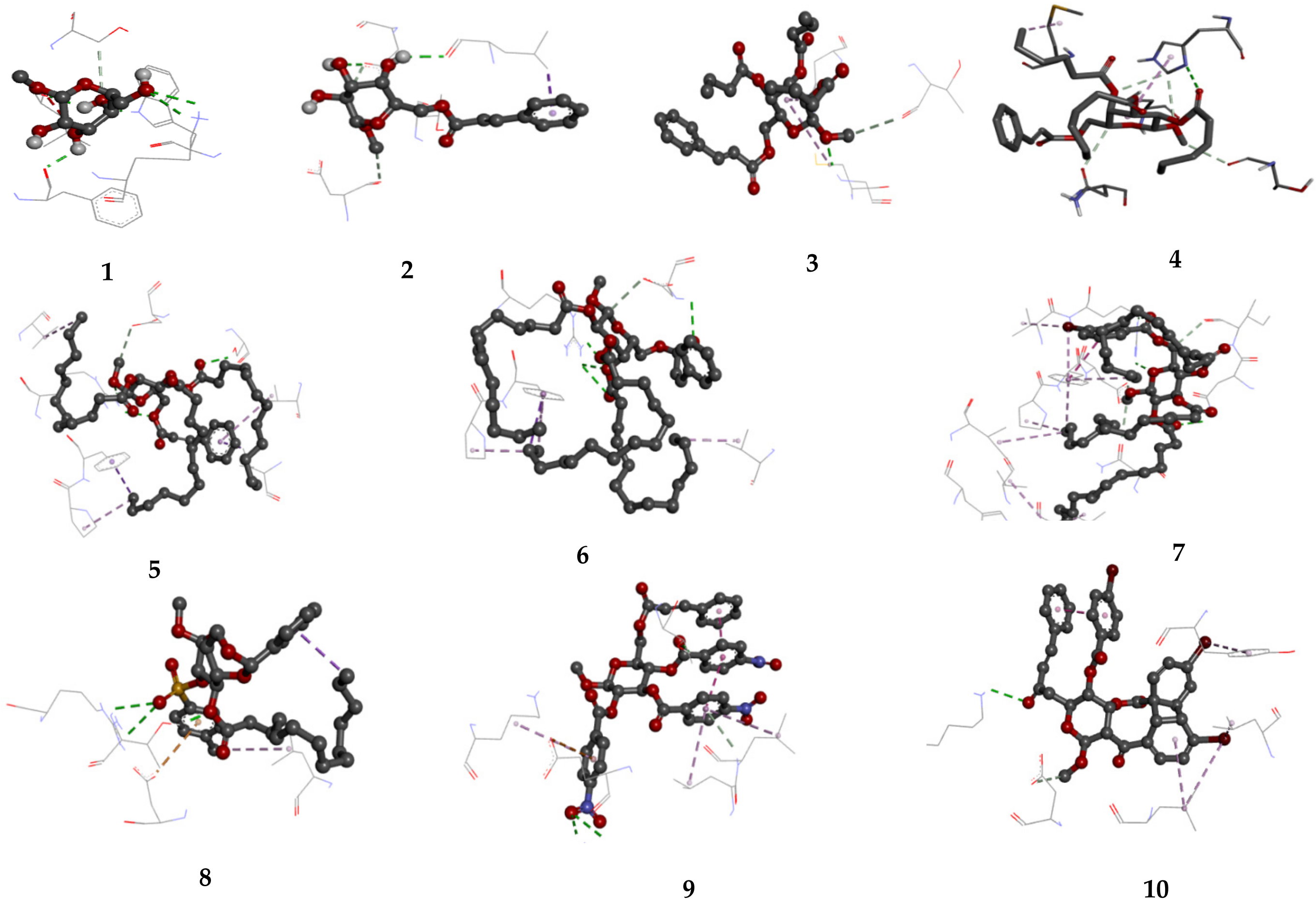

Figure S8. Protein-ligand interactions of all compounds.

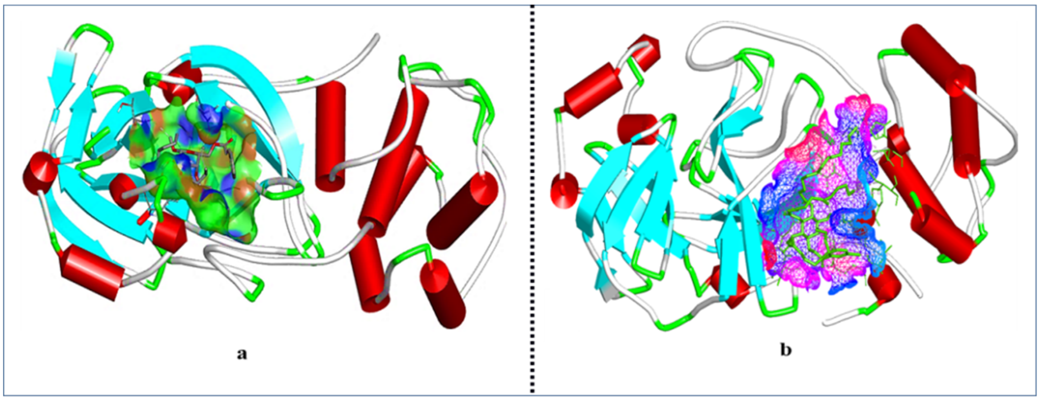

Figure S9. Hydrogen bond surface (**a**) and hydrophobic surface (**b**) of 6Y84 with analog (**7**).

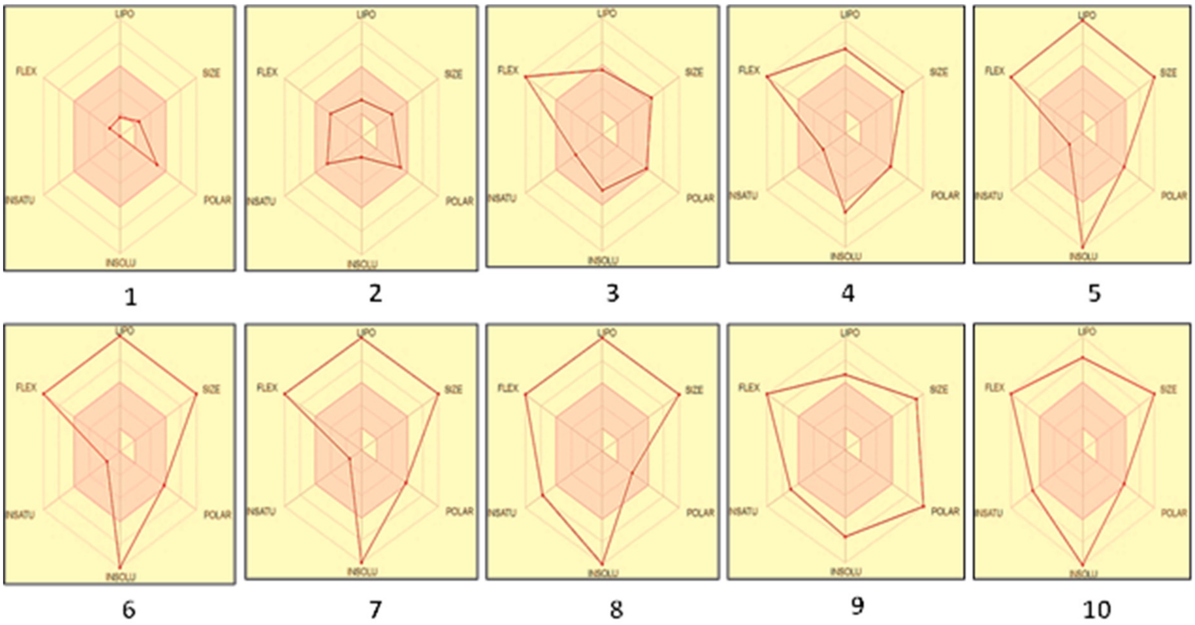

Figure S10. Bioactivity radar Charts of the MGP analogs where FLEX: Flexibility, LIPO: Lipophilicity, INSATU: Insaturation and INSOLU: Insolubility.

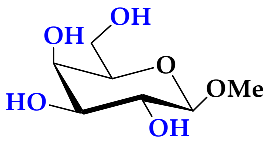

a

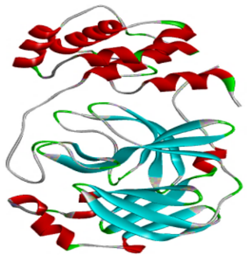

b

Figure S11. (**a**); 2D structure of MGP and (**b**); crystal structure of SARS-CoV-2 main protease (PDB: 6Y84).

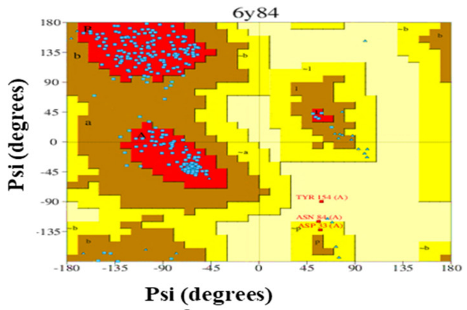

a

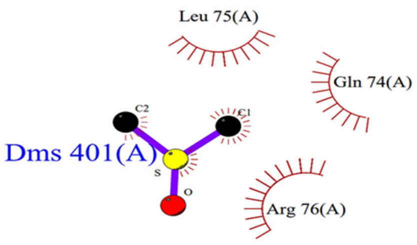

b

Figure S12. (**a**); Ramachandran plot for the SARS-CoV-2 main protease (PDB: 6Y84) (**b**); LigPlot image of the SARS-CoV-2 main protease (PDB: 6Y84) complex in 2D view predicted by PDBsum.

Table S1: Prediction of antimicrobial activity of the MGP analogs using PASS.

**Biological Activity**								
**Compound no.**	**Antibacterial**		**Antifungal**		**Antioxidant**		**Anti-carcinogenic**	
	**Pa**	**Pi**	**Pa**	**Pi**	**Pa**	**Pi**	**Pa**	**Pi**
**1**	0.541	0.013	0.628	0.016	0.403	0.041	0.731	0.008
**2**	0.530	0.014	0.672	0.011	0.647	0.004	0.834	0.004
**3**	0.549	0.012	0.702	0.010	0.518	0.006	0.737	0.007
**4**	0.551	0.012	0.713	0.009	0.515	0.006	0.739	0.007
**5**	0.551	0.012	0.713	0.009	0.515	0.006	0.739	0.007
**6**	0.551	0.012	0.713	0.009	0.515	0.006	0.739	0.007
**7**	0.551	0.012	0.713	0.009	0.515	0.006	0.739	0.007
**8**	0.433	0.024	0.612	0.017	0.506	0.006	0.578	0.014
**9**	0.550	0.017	0.654	0.013	0.478	0.008	0.644	0.011
**10**	0.520	0.015	0.657	0.013	0.488	0.007	0.598	0.013

Table S2. Molecular formula, molecular weight, electronic energy (*E*), enthalpy (*H*), Gibb's free energy (*G*) in Hartree, and dipole moment (μ, Debye) of MGP analogs.

Compound no.	MF	MW	E	H	G	μ
**1**	$C_7H_{14}O_6$	194.18	–722.2093	–722.2084	–722.2608	4.7712
**2**	$C_{16}H_{20}O_7$	324.33	–1141.5888	–1141.5878	–1141.6641	4.2649
**3**	$C_{28}H_{38}O_{10}$	534.60	–1831.3718	–1831.3709	–1831.4881	5.9289
**4**	$C_{34}H_{50}O_{10}$	618.75	–2065.7868	–2065.7859	–2065.9235	5.7353
**5**	$C_{52}H_{86}O_{10}$	874.25	–2769.0296	–2769.0286	–2769.2223	4.8235
**6**	$C_{58}H_{98}O_{10}$	955.39	–3005.1369	–3005.1358	–3005.4269	3.1229
**7**	$C_{64}H_{110}O_{10}$	1039.55	–3418.2109	–3418.2103	–3418.8237	4.6329
**8**	$C_{73}H_{62}O_7$	1051.29	–3719.9182	–3719.9172	–3719.9961	5.2934
**9**	$C_{37}H_{29}O_{16}N_3$	771.64	–2778.8441	–2778.8432	–2778.9831	5.6946
**10**	$C_{37}H_{29}O_{10}Br_3$	873.33	–9702.7449	–9702.7440	–9702.8701	6.4697

Table S3. Energy (eV) of HOMO, LUMO, Gap (Δ), hardness (*η*), softness (*S*), and chemical potential (*μ*) of MGP analogs.

Compound no.	HOMO	LUMO	Gap (Δε)	*η*	*S*	*μ*
**1**	–6.1918	1.3761	7.5679	3.7839	0.2643	–2.4078
**2**	–6.5306	–1.8074	4.7232	2.3616	0.4234	–4.1690
**3**	–6.3169	–1.6378	4.6791	2.3395	0.4274	–3.9773
**4**	–6.8035	–2.1736	4.6299	2.3149	0.4319	–4.4885
**5**	–6.6112	–1.9140	4.6972	2.3486	0.4257	–4.2626
**6**	–6.4647	–1.7998	4.6649	2.3324	0.4287	–4.1322
**7**	–6.9234	–1.4123	5.5111	2.7555	0.3629	–4.1678
**8**	–6.3212	–1.8312	4.4900	2.2450	0.4454	–4.0762
**9**	–6.6925	–3.5391	3.1534	1.5767	0.6342	–5.1158
**10**	–5.9226	–2.2964	3.6262	1.8131	0.5515	–4.1095

Table S4. Prediction of in silico of metabolism of MGP analogs.

Compound no.	Cyp1A2	Cyp2C19	Cyp2D6	Cyp3A4
**1**	No	No	No	No
**2**	No	No	No	No
**3**	No	No	No	Yes
**4**	No	No	No	Yes
**5**	No	No	No	Yes
**6**	No	No	No	Yes
**7**	No	No	No	Yes
**8**	No	No	No	Yes
**9**	No	No	No	Yes
**10**	No	No	No	Yes

Table S5. Prediction in silico of toxicity of MGP analogs.

Compound no.	Ames toxicity	Herg1 inhibition	LD50	Skin sensitization
**1**	No	No	1.533	No
**2**	yes	No	2.471	No
**3**	No	No	2.564	No
**4**	No	No	2.337	No
**5**	No	No	2.410	No
**6**	No	No	2.452	No
**7**	No	No	2.472	No
**8**	No	No	2.482	No
**9**	No	No	2.485	No
**10**	No	No	2.657	No
